# Hormonal contraceptive exposure relates to changes in resting state functional connectivity of anterior cingulate cortex and amygdala

**DOI:** 10.3389/fendo.2023.1131995

**Published:** 2023-07-13

**Authors:** Esmeralda Hidalgo-Lopez, Isabel Noachtar, Belinda Pletzer

**Affiliations:** ^1^ Centre for Cognitive Neuroscience, University of Salzburg, Salzburg, Austria; ^2^ Department of Psychology, University of Salzburg, Salzburg, Austria

**Keywords:** resting state fMRI, brain connectivity, hormonal contraceptives, progestins, amygdala, anterior cingulate cortex

## Abstract

**Introduction:**

Hormonal contraceptives (HCs), nowadays one of the most used contraceptive methods, downregulate endogenous ovarian hormones, which have multiple plastic effects in the adult brain. HCs usually contain a synthetic estrogen, ethinyl-estradiol, and a synthetic progestin, which can be classified as androgenic or anti-androgenic, depending on their interaction with androgen receptors. Both the anterior cingulate cortex (ACC) and the amygdala express steroid receptors and have shown differential functionality depending on the hormonal status of the participant and the use of HC. In this work, we investigated for the first time the relationship between ACC and amygdala resting state functional connectivity (rs-FC) and HC use duration, while controlling for progestin androgenicity.

**Methods:**

A total of 231 healthy young women participated in five different magnetic resonance imaging studies and were included in the final analysis. The relation between HC use duration and (i) gray matter volume, (ii) fractional amplitude of low-frequency fluctuations, and (iii) seed-based connectivity during resting state in the amygdalae and ACC was investigated in this large sample of women.

**Results:**

In general, rs-FC of the amygdalae with frontal areas, and between the ACC and temporoparietal areas, decreased the longer the HC exposure and independently of the progestin’s androgenicity. The type of HC’s progestin did show a differential effect in the gray matter volume of left ACC and the connectivity between bilateral ACC and the right inferior frontal gyrus.

## 1 Introduction

Consistent evidence has demonstrated ongoing plastic changes in the adult brain, including those related to ovarian hormones (1, 2). From neurogenesis to remyelination, the neuroactive actions of ovarian hormones are vast and still not fully understood (1, 3). In humans, brain network dynamics are also affected by ovarian hormone fluctuations, and more importantly, these effects appear to be suppressed during the use of hormonal contraceptives (HCs) (4). While women experienced physiological fluctuations of ovarian hormones throughout their adulthood, HCs, nowadays one of the most used contraceptive methods (5), abolish this cycle. In general, HCs downregulate the hypothalamic–pituitary–gonadal axis, decreasing the endogenous ovarian hormones’ production and maintaining their levels low, comparable to the levels observed in naturally cycling women during menses (6). In general, the synthetic hormones’ bioavailability remains stable during the active HC use and prevents follicle growth and ovulation (7, 8). HCs usually contain a synthetic estrogen, ethinyl-estradiol, but on the side of synthetic progestins, the range of compounds is wider. The latter can be classified into androgenic or anti-androgenic, depending on their interaction with androgen receptors (9, 10). On the one hand, those progestins derived from 19-nortestosterone (i.e., norethindrone, desogestrel, gestodene, and norgestimate) can be classified as androgenic due to their rapid metabolism to levonorgestrel, which demonstrates agonistic binding to the androgen receptor (11). On the other hand, those progestins derived from spironolactone (i.e., drospirenone), or from 17-hydroxyprogesterone (i.e., chlormadinone acetate or cyproterone acetate), can be classified as anti-androgenic due to their antagonistic actions at the androgen receptor (12). Additionally, dienogest, although derived from 19-nortestosterone, also exhibits anti-androgenic activities (13).

Despite the sparsity of literature regarding HC effects on the female brain, animal research and a few human studies hint at structural (14, 15) and functional changes (see reviews by 16, 17) related to HC use. Among the most consistently reported brain areas in ovarian hormones research are those belonging to the salience network. This network, crucial for emotion processing, is of special interest given that adverse mood effects are the major reason to discontinue HC use (18, 19). Both the anterior cingulate cortex (ACC) and the amygdala, at the core of this network, express steroid receptors (1, 3), and have shown differential functionality depending on the hormonal status of the participant (20–22). ACC not only shows differences in activation across the menstrual cycle, but also has been reported to be thinner (23) and to increase its activation to emotional stimuli (24) in HC users compared to naturally cycling women. Likewise, amygdala structure (25) and response to emotional stimuli differs between naturally cycling women and HC users (26), and across the menstrual cycle (27, 28).

Resting state functional connectivity (rs-FC), assessed as the correlated activity of different brain areas in the absence of an explicit task, offers a valuable measure to understand the brain’s intrinsic network organization (29, 30). Nowadays, we can investigate changes in rs-FC to disentangle the dynamics of the healthy brain functioning. Within this framework, neuroimaging studies have shown differences in the intrinsic connectivity of the salience network across menstrual cycle phase, and between naturally cycling women and HC users (for a review, see 16). Across the natural menstrual cycle, rs-FC between the ACC with the middle frontal, superior temporal, transverse temporal, and postcentral gyri (31) increased during the luteal phase when progesterone levels are high (31). In a recent study, we also described increased effective connectivity from the middle frontal gyrus to the ACC, but decreased effective connectivity from the medial prefrontal cortex to the ACC during the luteal phase (22). However, decreased rs-FC between ACC, middle frontal gyrus, and cuneus has also been described for luteal woman (32). Also, during this phase, the amygdala rs-FC with the right middle frontal gyrus, superior frontal gyrus, and paracentral lobule has been reported to increase (31). During the late luteal phase, though, rs-FC between the left amygdala and left angular gyrus and posterior cingulate cortex was decreased compared to the mid-follicular phase (33).

Regarding the HC effects on rs-FC, Engman et al. (31) investigated changes in the ACC and amygdala connectivity in response to a levonorgestrel containing HC in a randomized, placebo-controlled trial. They observed an increased rs-FC of the right ACC with the precuneus and left superior frontal gyrus and a decreased rs-FC between the right amygdala and the left postcentral gyrus, during HC intake (31). Additionally, between-group comparisons revealed increased rs-FC between the left ACC and precuneus, and left amygdala and left postcentral gyrus and precuneus for the naturally cycling luteal women compared to the HC users (31). Active HC users have also shown an increased rs-FC of the ACC within the salience network compared to naturally cycling women (34), but decreased rs-FC between the salience network and the left middle frontal gyrus, compared to both HC inactive phase and naturally cycling women (32). Lisofsky et al. (25) followed women before and after the start of HC use and reported decreased rs-FC between the left amygdala and the right IFG. However, these latter studies did not control for the androgenicity of the HC used, and it remains unclear whether androgenic and anti-androgenic HCs differentially modulate rs-FC of the salience network. Given that exogenous testosterone in women decreases connectivity between ACC and the inferior frontal gyrus (35), and between amygdala and the orbitofrontal cortex (36), it seems relevant to account for the type of progestin that the HCs contain. In this work, we investigated for the first time the relationship between ACC/amygdala rs-FC and HC use, while controlling for the progestin’s androgenicity.

Furthermore, while Engman et al. (31) investigated the effects of short-term HC-use (one intake cycle) on rs-FC of the salience network nodes, Petersen et al. (32) included long-term HC users. Given that results differ between the two studies, it is an interesting question whether changes in rs-FC of the salience network accumulate over time. Previous animal research has shown cumulative effects of sex steroids on the brain substrate, ranging from molecular to cellular levels (see reviews: 3, 37–40). In rodents, DNA de-methylation through the estrogen receptor is time-dependent (38), while continuous vs. sequential administration of progesterone elicits a differential gene expression profile in the hippocampus (40). After neural damage, progesterone-dependent recovery is affected by treatment duration, alongside differential effects on the cytoarchitectural structure (39). Relatedly, time-dependent effects on synaptic transmission are mediated by changes in shape and density of dendrites (39) and neurotransmitter’s receptors (41). Other steroids’ cumulative effects found in rodents include changes in the glial cells and consequent myelination, with an important role in neuroprotection (37, 42). Finally, and given that the reversibility of HC effects on the brain is still in question, we further explored if the effects found in HC users were replicated in previous HC users. Accordingly, we here opted for studying in a large sample of women those time-dependent associations that may accumulate over HC use duration, differentiating between androgenic and anti-androgenic progestins. In addition to addressing the temporal dynamics of HC effect or rs-FC of the salience network, the pattern of associations might shed some light into the endocrinological mechanisms underlying these effects. At the moment, it remains unclear whether changes in rs-FC during HC use are related to the progestagenic or androgenic/anti-androgenic actions of the progestin component, estrogenic actions of ethinyl-estradiol, or a by-product of HC effects on endogenous neurosteroids.

Given that the contraceptive effects of HC depend largely on the progestin component, the focus with regard to neuroplastic changes in response to HC is usually on the activation of progesterone receptors. Synthetic progestins possess a higher binding affinity for intracellular progesterone receptors than the endogenous hormone (43). If cumulative effects of HC on the salience network depend on progestagenic actions, we can expect effects as observed in the luteal phase (e.g., increased connectivity of the ACC and amygdalae to the middle frontal gyrus; 22, 31) to increase with increasing use duration, irrespective of the androgenicity of the progestin. Furthermore, one important consideration is that most of the effects of endogenous progesterone are exerted through its metabolite allopregnanolone. Synthetic progestins, however, are not metabolized to allopregnanolone, which is a potent modulator of one of the receptors for inhibitory neurotransmitter γ-aminobutyric acid (GABA) (for review, see 40). Although women under HC have increased synthetic progestin levels with high affinity for progesterone receptors, their circulating allopregnanolone levels seem to be decreased (androgenic HC: 44; anti-androgenic HC: 45). Accordingly, if previously reported effects along the natural menstrual cycle were related to endogenous allopregnanolone rather than actions on the progesterone receptor, we expect longer HC duration, related to decreased ACC connectivity with the temporal lobe, middle frontal and postcentral gyri connectivity (22, 31) while increased ACC connectivity with the medial prefrontal cortex (22). Likewise, for the amygdala, decreased connectivity with the middle, superior frontal gyrus and paracentral lobule (31) would be expected. These hypotheses are in line with results obtained from long-term HC users in previous studies (32). On the other hand, animal research hints a more complex scenario, in which allopregnanolone levels depend on the type of progestin (see systematic review, 46). In general, while androgenic progestins decrease allopregnanolone levels in the brain; anti-androgenic progestins increase them. If this is also the case in humans, further differential effects are expected for androgenic vs. anti-androgenic HCs related to levels of allopregnanolone.

More importantly, related to the differential modulation of androgen receptors by different progestins, opposite associations to the duration of androgenic vs. anti-androgenic progestin use can be expected. Although we would expect androgenic HC to have similar effects as testosterone (e.g., decreased ACC–inferior frontal gyrus and amygdala–orbitofrontal cortex connectivity; 35, 36), it is noteworthy that endogenous testosterone is metabolized to estradiol, acting also on estrogen receptors (47). Regarding estrogenic effects on the salience network, higher rs-FC between the amygdala and prefrontal and temporal areas has been reported after an estradiol challenge in postmenopausal women (48). Within naturally cycling women, increased connectivity between the amygdala and cuneus, inferior frontal fyrus, precentral gyrus, supramarginal gyrus, and temporal lobe has been described in the presence of high estradiol levels, while increased connectivity between amygdalae and the ACC has been described for low estradiol levels (49). Accordingly, if cumulative HC effects on the salience network are derived from the estrogenic actions of ethinyl-estradiol, we expect connectivity between the amygdalae and fronto-temporal areas to increase with use duration irrespective of the androgenicity of the progestin.

In general, a decreased rs-FC of the ROIs with frontal areas like middle frontal gyrus and inferior frontal gyrus is expected, related to longer HC exposure (25, 32). We also expect an increased rs-FC of the ACC with the precuneus and left superior frontal gyrus and a decreased rs-FC between the amygdalae and the left postcentral gyrus and precuneus, especially for HC with androgenic progestins (31). Further differential effects of androgenic vs. anti-androgenic HC will be explored.

## 2 Methods

### 2.1 Participants and procedure

A total of 231 healthy young women (105 current HC users and 126 past HC users and currently naturally cycling) were included in the final analysis, from five different MRI studies (50–52). Participants were recruited via flyers at the University of Salzburg and via online advertisements. Most of the participants were university students and all of them were right-handed. Main exclusionary criteria for women were neurological, psychiatric, or endocrine disorders, any medication intake, or any brain abnormalities displayed on structural MRI. Note that differential brain organization may underlie women’s susceptibility to HC adverse mood side effects, which is the major reason for HC discontinuation (19, 53).

In all studies, scanning sessions were scheduled in the active phase of HC use for current HC users (second or third week of the intake cycle), or locked to their menstrual cycle for naturally cycling women (past users). For the latter, the majority of sessions were scheduled during the early follicular phase (cycle days 1–8), except for six participants that were in their mid-luteal phase (3–10 days before onset of next menses). Participants had to confirm the onset of next menses in retrospect.

As part of our standard screening questionnaire, information on previous contraceptive use was collected. The participants were subdivided into HC users with current androgenic HC use (A-HC; *n* = 62) and current anti-androgenic HC use (AA-HC; *n* = 43), and naturally cycling women with previous androgenic HC use (A-NC; *n* = 45), previous anti-androgenic HC use (AA-NC; *n* = 52), and unknown androgenicity of the HC (*n* = 29). Further details regarding the categorization of progestins into androgenic or anti-androgenic and the subsample size of each specific progestogenic component can be found in the **Supplementary Material**.

Only women currently or previously using HCs with only one and the same type of progestin (either A or AA) were included. Specifically for naturally cycling women, only participants who had not been using any HCs or IUDs for the past 6 months and had a regular menstrual cycle, defined as between 21 and 35 days, and less than 7 days of cycle length variability (54), were included. All participants gave their signed written consent to participate in each study. Every study was approved by the University of Salzburg’s ethics committee and conforms to the Code of Ethics of the World Medical Association (Declaration of Helsinki).

The androgenicity of HC was coded as a categorical variable with the following levels: level “A”: androgenic, level “AA”: anti-androgenic, and level “unknown”. Further details are described in the following paragraphs. In order to compare age and HC use duration between androgenic and anti-androgenic users, independent-samples *t*-test was performed. For women currently using HC, there was a significant difference in age between A-HC (M = 21.31, SD = 2.55) and AA-HC (M = 22.58, SD = 3.57); *t*_(103)_ = −2.13, *p* = 0.04. Anti-androgenic users were, on average, approximately 1 year older than androgenic users. Likewise, use duration was significantly different between A-HC (M = 4.00, SD = 2.47) and AA-HC (M = 5.12, SD = 2.92); *t*(103) = −2.12, *p* = 0.04. The HC use duration for anti-androgenic users was, on average, approximately 1 year longer than the androgenic duration. For the naturally cycling women, age and HC use duration were not significantly different between women who had previously taken androgenic or anti-androgenic HC (*t* > |2.5|, *p* > 0.05) (**Table 1**). HC use duration was not related to ethinyl-estradiol levels (*r* < 0.01, *p* = 0.23), and the distribution of HC use duration by each different type of progestin followed similar distributions.

**Table 1:** Participants’ demographics.

	Current HC users				Past HC users				
Type of scanner	TRIO		PRISMA		TRIO			PRISMA	
Type of progestin	A	AA	A	AA	A	AA	Unknown	A	AA
Sample size (*n*)	32	20	30	23	24	32	29	21	20
HC use duration in years M (SD)	4.71 (2.80)	4.89 (2.34)	3.25 (1.82)	5.32 (3.39)	4.24 (3.00)	4.65 (4.33)	2.09 (2.00)	3.97 (3.01)	3.80 (3.50)
Age M (SD)	22.53 (2.77)	21.80 (2.84)	20.00 (1.44)	23.26 (4.05)	25.96 (4.62)	24.47 (3.72)	25.45 (5.51)	24.24 (3.32)	25.00 (4.00)

HC, hormonal contraceptive; A, androgenic; AA, anti-androgenic; M, mean; SD, standard deviation.

In order to control for possible moderation effects of ethinyl-estradiol levels and given previously reported dose-dependent cognitive effects (55), the following analyses were further performed controlling for the levels present in the HC. No interactive effect of pill duration by ethinyl-estradiol levels was observed, and therefore, the rest of the analyses and results will focus on the main effect of HC use duration and interaction with HC androgenicity.

### 2.2 fMRI data acquisition

For each study, a resting state scan of approximately 9 min duration was performed at the beginning of the scanning session. Participants were instructed to close their eyes, relax, and let their mind wander. One of the studies, however, instructed the participants to leave their eyes open (HC = 20, 19% of the final sample; NC = 28, 22% of the final sample). When the sample from this study was excluded, partial correlations between HC use duration and brain connectivity effects remained significant (*pr*_age, type of scanner_ > |0.35|, *p* < 0.05). Therefore, participants from all five studies were included in the following analyses. Two types of scanners were used: a Siemens Magnetom TIM Trio 3 Tesla and a Siemens Magnetom Prisma Fit 3 Tesla, both of them with a 64ch head coil (see **Table 1** for demographics). Although we add a regressor for potential confounding effects of the type of scanner in all of the analyses, the combination of these datasets still poses a conceptual limitation. The impact of Siemens Tim Trio to Prisma upgrade has been assessed in different quantitative MRI measures (56) and proton magnetic resonance spectroscopy (57). Higher reliability was identified in most of the MRI outputs that were investigated across the Prisma upgrade (56) and inter-scanner variation had average values of approximately 2.2%–3.8% (56). Although there is also a fair level of intra-vendor consistency in individual scans (58), the present datasets also differed in their respective sequences’ TR. Therefore, we explored additional interactions of the type of scanner with the HC used duration effects herein presented. No significant interactive effects were found.

For the first four studies, functional and high-resolution structural images were acquired on the TIM Trio scanner following a field map acquisition. Functional images consisted of a T2-weighted gradient echo planar sequence with 36 transversal slices oriented parallel to the AC-PC line (whole-brain coverage, TE = 30 ms, TR = 2,250 ms, flip angle 70°, slice thickness 3.0 mm, matrix 192 × 192, FOV 192 mm). For structural images, we acquired a T1-weigthed 3D MPRAGE sequence (160 sagittal slices, slice thickness = 1 mm, TE = 2.91 ms, TR = 2,300 ms, TI delay 900 ms, flip angle 9°, FOV 256 × 256 mm). For the most recent study, functional and high-resolution structural images were acquired on the same MRI device, upgraded to the Prisma Fit system. Functional images consisted of a T2-weighted gradient echo planar sequence with 64 transversal slices oriented parallel to the AC-PC line (whole-brain coverage, multi-slice interleaved, TE = 30 ms, TR = 1,400 ms, flip angle 69°, slice thickness 2.3 mm, matrix 202 × 202, FOV 202 mm). For structural images, we acquired a T1-weigthed 3D MPRAGE sequence (176 sagittal slices, slice thickness = 1 mm, TE = 2.91 ms, TR = 2,300 ms, TI delay 900 ms, flip angle 9°, FOV 256 × 256 mm). In the Prisma scanner study, in order to create unwrapped field maps that can be used to do B0 inhomogeneity distortion correction of the functional scans, two echo planar images (EPI) with opposite phase encode directions were acquired right before the resting state.

### 2.3 fMRI data analyses

For functional images, the first six images of each session were discarded. The remaining scans were despiked using 3d-despiking as implemented in AFNI (afni.nimh.nih.gov). The resulting images were pre-processed using SPM12 standard procedures and templates including (i) realignment and unwarping of the functional images using the field map, (ii) segmentation of the structural images using CAT12, (iii) co-registration of the functional images to the structural images, (iv) normalization of functional images using the normalization parameters as estimated by CAT12, and (v) spatial smoothing using a 6-mm kernel. Additionally, for the Prisma scanner, the field map was calculated from the two EPI images with opposite phase encoding using the FSL “topup” tool (http://fsl.fmrib.ox.ac.uk/fsl/fslwiki/TOPUP; 59), and the Field Map Toolbox from SPM12 was used to calculate a voxel displacement map to correct the BOLD EPI images (https://lcni.uoregon.edu/kb-articles/kb-0003; 60). Pre-processing quality control procedures included the automatic exclusion of participants with excessive movement (>3 mm translation, >2° rotation), visual inspection of structural and functional scans ensuring adequate coregistration, and visually checking the normalization to a standard T1 and an EPI MNI template. Finally, we perform ICA-AROMA non-aggressive removal of artifactual components on the resulting images. ICA-AROMA has been shown to reduce motion-induced variation in fMRI signal, while preserving the signal of interest (61).

#### 2.3.1 Gray matter volume and fractional amplitude of low-frequency fluctuations

Gray matter volumes from bilateral ACC and amygdala were extracted using the get_totals script by G. Ridgeway (http://www0.cs.ucl.ac.uk/staff/gridgway/vbm/get_totals.m), each region of interest (ROI) defined with the AAL atlas (62).

The fractional amplitude of low-frequency fluctuation (fALFF) maps were calculated from pre-processed resting state images using the DPABI toolbox (63). The fALFF is a measure of oscillatory activity at the resting state, relative to the whole frequency range (64). It is defined as the ratio of the power spectrum of low frequency (0.01–0.08 Hz) to the average square root of each frequencies power within this range (64, 65).

In order to assess the duration of the HC use*androgenicity interactive effect on (i) gray matter volume and (ii) fALFF, they were introduced as dependent variables in linear models in R Version 1.4.1717, using the lm function from the stats package (66). For all models, HC use duration, androgenicity, and their interaction were included as fixed effects. In case no significant interaction between androgenicity and HC use duration was observed, the interaction was removed from the model and the main effect of HC use duration was calculated across both groups. For every model, age and type of scanner were added as nuisance regressors (e.g., fALFF ~ duration of HC use*androgenicity + age + type of scanner). For the models including gray matter volume, we used the total intracranial volume (TIV) as an additional covariate (e.g., GM IFG ~ duration of HC use*androgenicity +TIV + age + type of scanner). All continuous variables were scaled prior to analyses to allow for interpretation of effect sizes based on standard deviations.

#### 2.3.2 Seed-to-voxel connectivity analysis

We investigated bilateral ACC and amygdala functional connectivity with AAL atlas-defined region of interest (ROI) (62). Seed-to-voxel connectivity maps from these ROIs were estimated for each subject using the CONN-toolbox standard procedures and templates (67). The six movement parameters as well as five white matter and cerebrospinal fluid components were used as regressors during the denoising step. A band-pass filter of 0.008–0.09 Hz was applied. For the group-level analysis, full factorial models were used to evaluate the overall connectivity relation to HC use duration. For each of the ROIs, the first-level contrast images were introduced into a full factorial design in order to investigate the interactive effect of duration of HC use and HC group (androgenic vs. anti-androgenic). In case no significant interaction between androgenicity and HC duration was observed, the main effect of HC use duration was calculated across both groups. In order to control for age and scanning upgrade, their interaction with the HC group was additionally modeled as nuisance regressors. For this second level, results were masked with an SPM gray matter template, and we used an extent threshold of *k* = 20 voxels, an uncorrected primary threshold of *p* < 0.001, and a secondary cluster-level FWE-corrected threshold of *p* < 0.05 (indicated as pFWE). In case a cluster of significant interaction between androgenicity and HC use duration emerged, eigenvalues were extracted from this cluster and partial correlations controlling for age and type of scanner were performed separately for androgenic and anti-androgenic HC users. In a follow-up analysis, we checked if the effects in HC users were replicated in naturally cycling previous HC users. The first eigenvector of the time series across voxels was extracted from the significant FWE-corrected clusters found in HC current users, and partial correlations for naturally cycling previous HC users were performed controlling for both age and type of scanner. In case of no significant interaction between androgenicity and HC use duration, the level “unknown” from HC androgenicity variable was included in the partial correlation. Otherwise, only known “A” and “AA” previous HC users were included and partial correlations were explored separately.

## 3 Results

### 3.1 Gray matter volume and fractional amplitude of low-frequency fluctuations

For the gray matter volume of the ROIs, there was an interactive effect of HC use duration and androgenicity in the left ACC [*b* = −0.31, SE_b_ = 0.15, *t*_(98)_ = −1.99, *p* = 0.049]. Current androgenic users showed a smaller GM volume of the left ACC the longer the duration of HC use (**Figure S1**). However, partial correlations were separated by androgenicity, and controlling for age, TIV and scanner type did not survive the significance threshold [*pr*_age, type of scanner, TIV_ = −0.05, *p*_(57)_ = 0.73 for A-HC, and *pr*_age, type of scanner, TIV_ = −0.23, *p*_(38)_ = 0.15 for AA-HC].

No further significant relations were found for right ACC or bilateral amygdala gray matter volume. Neither ACC nor amygdala showed any previous HC use duration effect [all *pr*_age, type of scanner_ <|0.015|, *p*_(122)_ > 0.05].

No significant relations were found for bilateral ACC or amygdala between fALFF and duration of HC use.

### 3.2 Seed-to-voxel connectivity analysis

Whole brain connectivity maps of bilateral ACC and amygdalae are displayed in the **Supplementary Material (S2–S5)**. For the ACC, positive connectivity maps included insular and medio-temporal areas, while negative connectivity maps included superior parietal lobes and inferior frontal gyri. Positive connectivity maps for the amygdalae included insular, middle cingulate, and ventromedial prefrontal cortices, among other medio-temporal areas, putamen, and thalamus. Negative connectivity maps for the amygdalae included superior, middle frontal, and angular gyri, among others.

#### 3.2.1 Main effect of HC use duration

**Figure 1:**
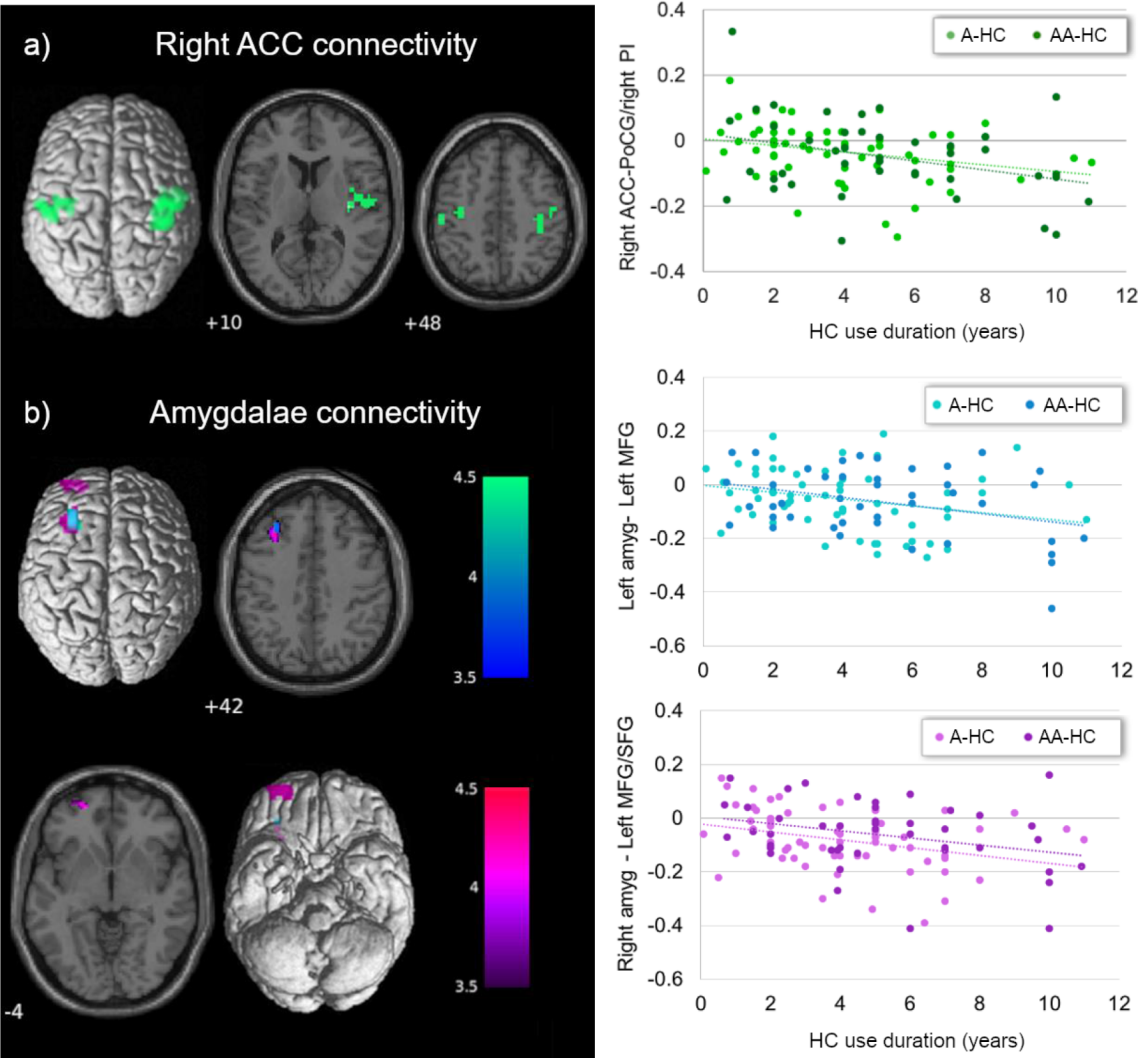
Main effect of current HC use duration on ACC and amygdala connectivity. **(A)** Current HC users showed lower connectivity between the right ACC and right insula/bilateral post-central gyrus, the longer the use of HC, irrespective of the androgenicity (in green). **(B)** Independently of the androgenicity of the HC, current users showed lower connectivity between left (in blue) and right (in purple) amygdala with left prefrontal cortex the longer the use of the HC. ACC, anterior cingulate cortex; PoCG, post-central gyrus; PI, posterior insula; amyg, amygdala; MFG, middle frontal gyrus; SFG, superior frontal gyrus; HC, hormonal contraceptive; A, androgenic; AA, anti-androgenic.

For the ACC connectivity, we observed an inversed main effect of current HC use duration and connectivity between the right ACC and left post-central gyrus ([-54, -22, 49], 79 voxels, *T* = 4.63, *p*_FWE_ = 0.001), right posterior insula ([36, -16, 10], 67 voxels, *T* = 4.58, *p*_FWE_ = 0.003), and right pre/post-central gyrus ([39, -16, 61], 121 voxels, *T* = 4.54, *p*_FWE_ < 0.001). Functional connectivity between the right ACC and these three clusters was lower the longer the use of HC in current users, irrespective of the androgenicity (**Figure 1A**). Connectivity between the right ACC and these three clusters of interest did not show any previous HC use duration effect [all *pr*_age, type of scanner"_ < |0.015|, *p*_(122)_ > 0.05].

We also observed an inversed main effect of HC use duration on connectivity between bilateral amygdalae and prefrontal cortex. Connectivity between bilateral amygdalae and the left middle frontal gyrus ([-30, 35, 40], 37 voxels, *T* = 3.79, *p*_FWE_ = 0.042, for the left amygdala; [-30, 26, 46], 62 voxels, *T* = 4.42, *p*_FWE_ = 0.004, for the right amygdala) and between the right amygdala and the ventral part of the superior frontal gyrus ([-21, 56, -5], 37 voxels, *T* = 4.47, *p*_FWE_ = 0.042) was lower the longer the duration of HC, irrespective of the androgenicity (**Figure 1B**). Only for the connectivity between the right amygdala and the left superior frontal gyrus did we observe a similar effect of previous HC use duration in naturally cycling participants [*pr*_age, type of scanner_ = −0.19, *p*_(122)_ = 0.035]. Independently of the androgenicity type, the connectivity between the right amygdala and the ventral part of the superior frontal gyrus ([-21, 56, -5], 37 voxels) was lower the longer the use of HC in previous users (**Figure S6**).

#### 3.2.2 Interactive effect of HC use duration and HC androgenicity

**Figure 2:**
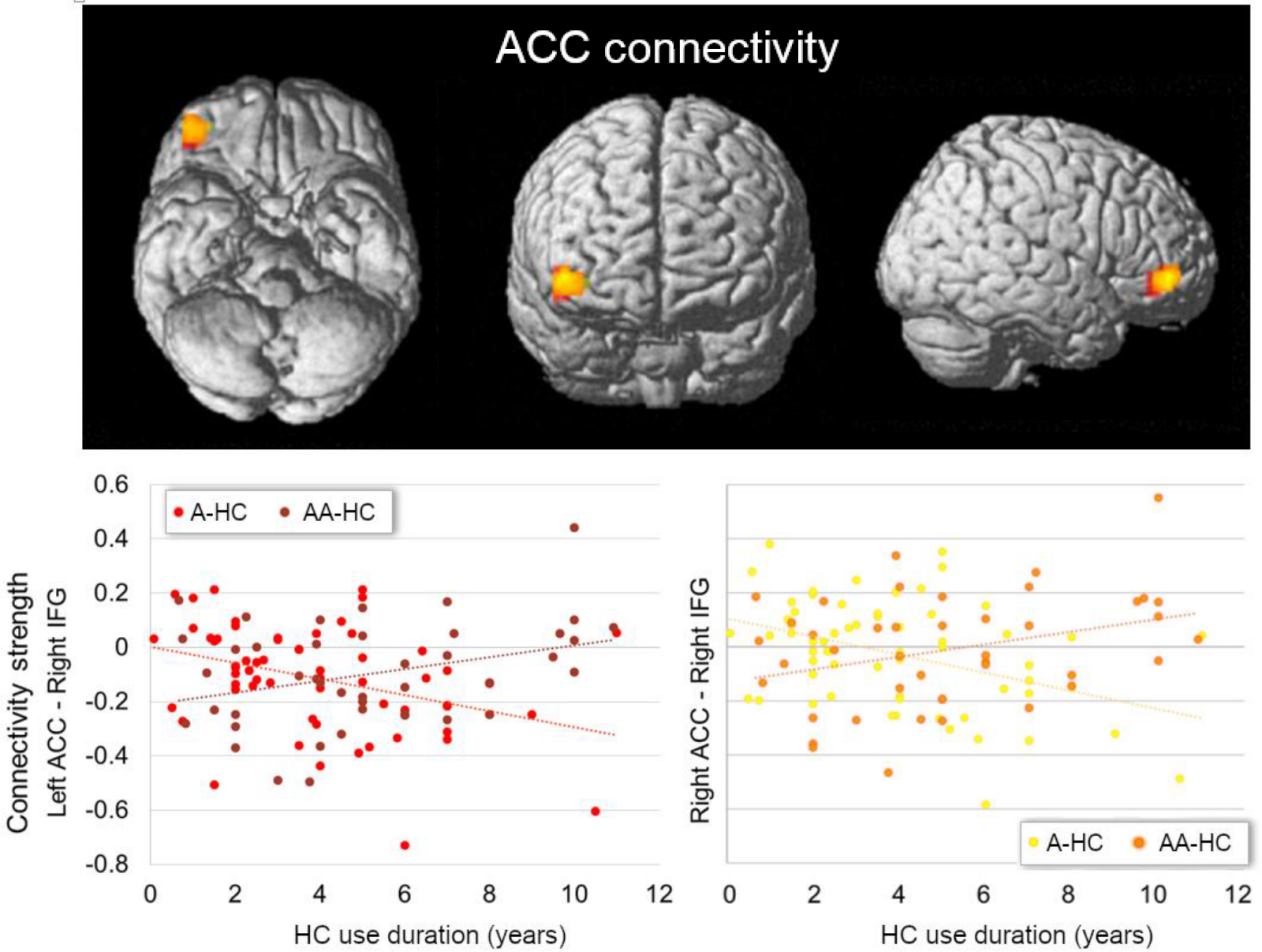
Interactive effect of current HC use duration and androgenicity on the connectivity between left (in red) and right ACC (in orange) with right inferior frontal gyrus (IFG). Current users of an androgenic HC (A-HC) showed lower connectivity between bilateral ACC and the right IFG the longer the HC use. In contrast, current users of an anti-androgenic HC (AA-HC) showed stronger connectivity between bilateral ACC and the right IFG the longer the HC use. ACC, anterior cingulate cortex; IFG, inferior frontal gyrus HC, hormonal contraceptive; A, androgenic; AA, anti-androgenic.

We further observed an interactive effect of HC use duration and androgenicity in the connectivity between left and right ACC with the triangular part of the right inferior frontal gyrus ([45, 47, -2], 50 voxels, *T* = 4.18, *p*_FWE_ = 0.012, for the left ACC; [39, 47, -5], 39 voxels, *T* = 4.35, *p*_FWE_ = 0.038, for the right ACC). For current users of an androgenic HC, the connectivity between bilateral ACC and the right inferior frontal gyrus was lower the longer the HC use [*pr*_age, type of scanner_ = −0.46, *p*_(58)_ < 0.001 for the left ACC; *pr*_age, type of scanner_ = −0.44, *p*_(58)_ < 0.001 for the right ACC]; for anti-androgenic HC users, connectivity strength was higher the longer the HC use [*pr*_age, type of scanner_ = 0.34, *p*_(39)_ = 0.03 for the left ACC; *pr*_age, type of scanner_ = 0.31, *p*_(39)_ = 0.04 for the right ACC; **Figure 2**]. Connectivity between the ACC and inferior frontal gyrus did not show any previous HC use duration effect in naturally cycling participants [all *pr*_age, type of scanner_ < |0.015|, *p*_(122)_ > 0.05]. No interactive effect of androgenicity and HC use duration was observed for the rs-FC of the amygdalae.

In summary, for the current HC users, the connectivity between the right ACC and right insula/bilateral post-central gyrus, and between bilateral amygdalae and left prefrontal cortex, was lower the longer the use of HC, irrespective of the androgenicity. For androgenic HC users, the connectivity between bilateral ACC and the right IFG was lower the longer the HC use; for anti-androgenic HC users, connectivity strength was higher the longer the HC use.

## 4 Discussion

In this study, we investigated for the first time the differences in the resting state functional connectivity (rs-FC) network of the anterior cingulate cortices (ACC) and the amygdalae related to the duration of androgenic (A) or anti-androgenic (AA) HC use. In general, rs-FC of the ACC and temporoparietal areas, and between the amygdalae with frontal areas, decreased the longer the HC exposure and independently of the progestin androgenicity. Androgenicity did show a differential effect in the connectivity between bilateral ACC and the right inferior frontal gyrus (IFG).

Longer HC use duration, irrespective of the androgenicity, was related to decreased connectivity between the ACC and the insular cortex, both involved in the salience network. Although naturally cycling women show within salience network connectivity increased related to enhanced endogenous progesterone (22, 53), this effect was absent in women during HC use (53). The impact of HC on salience-dependent processes such as motivation and reward-oriented behavior has been related to a decrease of the insula activation, for example, to sexual cues (68). In animal models, this sexual behavior has been shown to be impaired by exogenous hormonal treatment, changes suggested to be mediated by the blunted levels of allopregnanolone (69). Conversely, Sharma et al. (34) reported an increased salience within-network connectivity with the medial superior frontal gyrus for the HC users compared to the naturally cycling women (34). It needs to be noted, though, that pubertal-onset HC users were included in this comparison and given that this sub-group was reported to show significantly general increased connectivity compared to the adult-onset sub-group, it is unclear to which extent they drove the direction of the results.

Contrary to our expectations, we did not find a decreased connectivity between the amygdala and the postcentral gyrus and precuneus (31). Instead, it was the ACC that showed decreased connectivity to the bilateral pre/postcentral gyri the longer the HC use. In a recent study analyzing effective connectivity in a placebo-controlled trial, connections between the dorsal ACC and parietal areas decreased during androgenic HC treatment (53). In naturally cycling women, connectivity between the ACC and postcentral gyrus increased during the luteal phase (31), and the connectivity of the somatosensorial cortices was also positively related to progesterone levels (70). Connectivity between amygdalae with superior and middle frontal gyrus was also found decreased the longer the HC use in the present sample, in line with Petersen et al. (32). Moreover, although it did not survive the FWE correction in the whole-brain analysis, the right amygdala also showed reduced connectivity with the ipsilateral middle frontal gyrus, the longer the HC use duration (see **Supplementary Material**, **Figure S7**). Conversely, increased connectivity between these areas has been reported before during the progesterone-dominated luteal phase in naturally cycling women (31).

Following the argument that while HC women have increased synthetic progestin levels, their allopregnanolone levels remain decreased (44, 45), and that some effects of endogenous progesterone are exerted through this metabolite, these changes in opposite directions may suggest that the ACC-insular/somatosensorial and prefrontal–amygdalar connectivity positively relates to allopregnanolone levels, which, in turn, remains decreased during HC use. Relatedly, exogenous administration of progesterone, which significantly increased allopregnanolone levels, selectively increased amygdala reactivity (71). However, although animal research corroborates these effects for androgenic progestins, except for drospirenone (72), anti-androgenic progestins appear to increase allopregnanolone levels in the rodent brain (73, 74). On the other hand, although ethinyl-estradiol has a greater affinity for estrogen receptors than endogenous estradiol (75), it is administered in a much lower dose and also shows a differential selectivity for the alpha receptor type over the beta receptor type (76). The present findings could be a consequence of the cumulative effect of synthetic hormones, the abolishment of cyclic endogenous hormonal fluctuations, or the combination of both.

Contrary to our hypotheses, we only found an interactive effect of androgenicity and use duration for bilateral ACC. The connectivity of these areas with the right inferior frontal gyrus was lower the longer the androgenic HC use, while it was stronger the longer the anti-androgenic HC use. In post-menopausal women, connectivity between these areas during memory tasks is positively related to estradiol levels (77). Conversely, in pre-menopausal women using HC, bilateral ACC–inferior frontal gyrus connectivity has been reported to decrease after testosterone administration, during an empathy-related task (35). In male patients, anabolic androgen users showed decreased dorsal attention network connectivity with superior and inferior frontal gyri (SFG/IFG) and the ACC, related to use duration (78). Opposed effects of HC depending on the androgenicity of their progestin have also been extended to the behavioral level by some studies (see review, 79). Although these findings indicate opposite cumulative effects of androgenic vs. estrogenic modulation, both androgenic and anti-androgenic progestins reduce overall testosterone bioavailability (80, 81). Therefore, HC’s androgenicity impact needs to be further elucidated, preferably in longitudinal placebo-controlled trials.

Most of the effects observed in current HC users did not replicate for previous HC users, which could be interpreted as reversibility for such effects. Only the connectivity between the right amygdala and the ventral area of the left superior frontal gyrus still showed a decrease the longer the duration of previous HC use. We have previously described an effect of HC exposure on gray matter volume of subcortical structures, some of which also appear to remain after discontinuation (82). Although some studies hint at a chronic decrease in endogenous hormone levels many years after cessation of HC use (83), and long-term effects in task performance (84), the extent to which fronto-amygdalar connectivity is directly influenced by a prolonged HC use is still undetermined. Further longitudinal randomized placebo-controlled studies need to be carried out in order to fully disentangle the causal effect of HC and its reversibility after discontinuation.

Some remarks and limitations need to be noted. First, and important for the interpretations of these results, is that while a cross-sectional group comparison could identify those effects that emerge after the first months of use, but do not accumulate over time, here we investigated time-dependent associations. Therefore, for those results conflicting with previous literature, an alternative explanation is that following this early impact (that would explain the group differences), the changes adapt/regress over time. Further inconsistencies could also be partly attributable to the small sample and effect sizes of past studies. Second, although the categorization of progestins used here corresponds to their androgenic vs. anti-androgenic effects, this does not always reflect the corresponding androgenicity of the HCs. Progestins with a stronger androgenic effect may be found in lower doses in the HC and therefore have lower androgenic effects in the body once dosage is taken into account (85).Third, HC-related differences could be modulated by a continued suppression of the endogenous hormones, by the exposure to synthetic hormones, and/or by the interaction of both effects. As previously described, endogenous estradiol has a lower affinity for estrogen receptors than ethinyl-estradiol, and endogenous progesterone also differs to the different types of synthetic progestins in their extra-progestogenic effects. For example, they present a different affinity and agonist or antagonist modulation of gluco- and mineralocorticoid receptors (86). Furthermore, the involvement of these receptors in the regulation of the stress response is of special importance when considering potential long-term effects of HC on the brain. Last, but not least, we selected the present ROIs based on previous literature and delimited by the AAL atlas (62) for replication purposes (31). However, there are conceptual and practical challenges when selecting a specific parcellation, including the lack of precision in terms of inter-individual homologous correspondence in brain cortex (87, 88). Additional bias towards smaller sub-networks instead of larger brain systems has also been suggested for seed-based analyses (89).

Overall, these results in a large sample of women suggest cumulative changes in functional connectivity patterns at rest related to the extent of exposure to HC and the abolishment of the endogenous fluctuation of ovarian hormones. Differential effects of the type of progestin arose for some of these functional changes. Given the widespread use of HC among women, and the early onset of HC use, usually starting during adolescence, elucidating the synthetic progestins effects and the functional implications of these findings is of the utmost importance.

## Data availability statement

Data and scripts are openly available at https://osf.io/5ezw9/. MR images are available upon request from the corresponding author.

## Ethics statement

Every study was approved by the University of Salzburg’s ethics committee and conforms to the Code of Ethics of the World Medical Association (Declaration of Helsinki). The patients/participants provided their written informed consent to participate in this study.

## Author contributions

BP designed and made the concept of the study. BP, EH-L, and IN were responsible for acquiring the data. EH-L was responsible for data curation and analysis, interpreting the results, and drafting and revising the manuscript. BP supervised the analysis, contributed in the results’ interpretation, and revised the manuscript. All authors contributed to the article and approved the submitted version.
