# Supplementary material for: Hormonal contraceptive exposure relates to changes in resting state functional connectivity of anterior cingulate cortex and amygdala
Source: Front Endocrinol (Lausanne). 2023 Jul 13;14:1131995. doi: 10.3389/fendo.2023.1131995 (PMC10374315; doi:10.3389/fendo.2023.1131995)
Supplement: Supplementary file 1 [file DataSheet_1.pdf]

Supplementary material

1. Methods

1.1. Participants and procedure

Subsamples distribution for each progestogenic component of HC:

HCs containing levonorgestrel (n_{HC}=41; n_{NC}=14), desogestrel/etonorgestrel (n_{HC}=8; n_{NC}=14), gestoden (n_{HC}=12; n_{NC}=12), norelgestromin/ norgestimate (n_{HC}=1; ; n_{NC}=3) and norethisterone (n_{NC}=2) were included in the type *androgenic;* and HCs containing drospirenone (n_{HC}=15; n_{NC}=22), chlormadinone acetate (n_{HC}=13; n_{NC}=12), dienogest (n_{HC}=7; n_{NC}=14), and cyproterone acetate (n_{HC}=8; n_{NC}=4), were included as *anti-androgenic*.

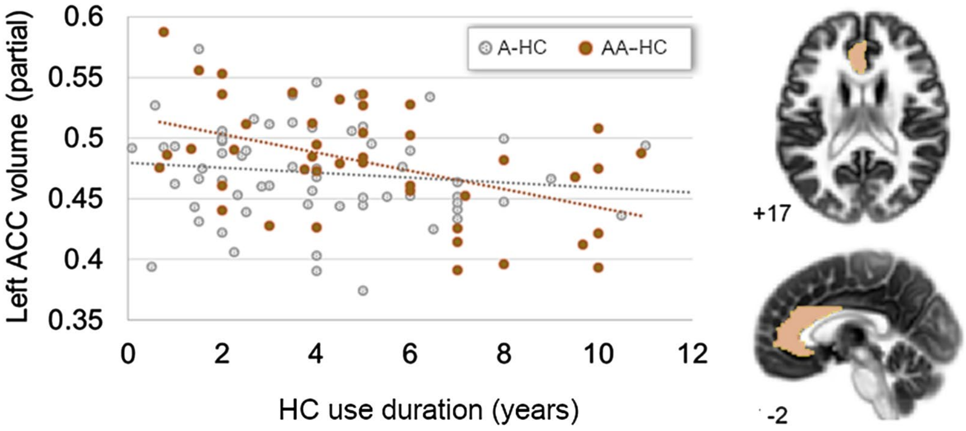

Figure S1. Interactive effect of current HC use duration and androgenicity on the left ACC GM volume. Current users of an anti-androgenic HC (AA- HC) showed lower GM volume in the left ACC the longer the HC use. This effect was not observed for the androgenic HC (A- HC) users. HC: hormonal contraceptive; A: androgenic; AA: anti-androgenic.

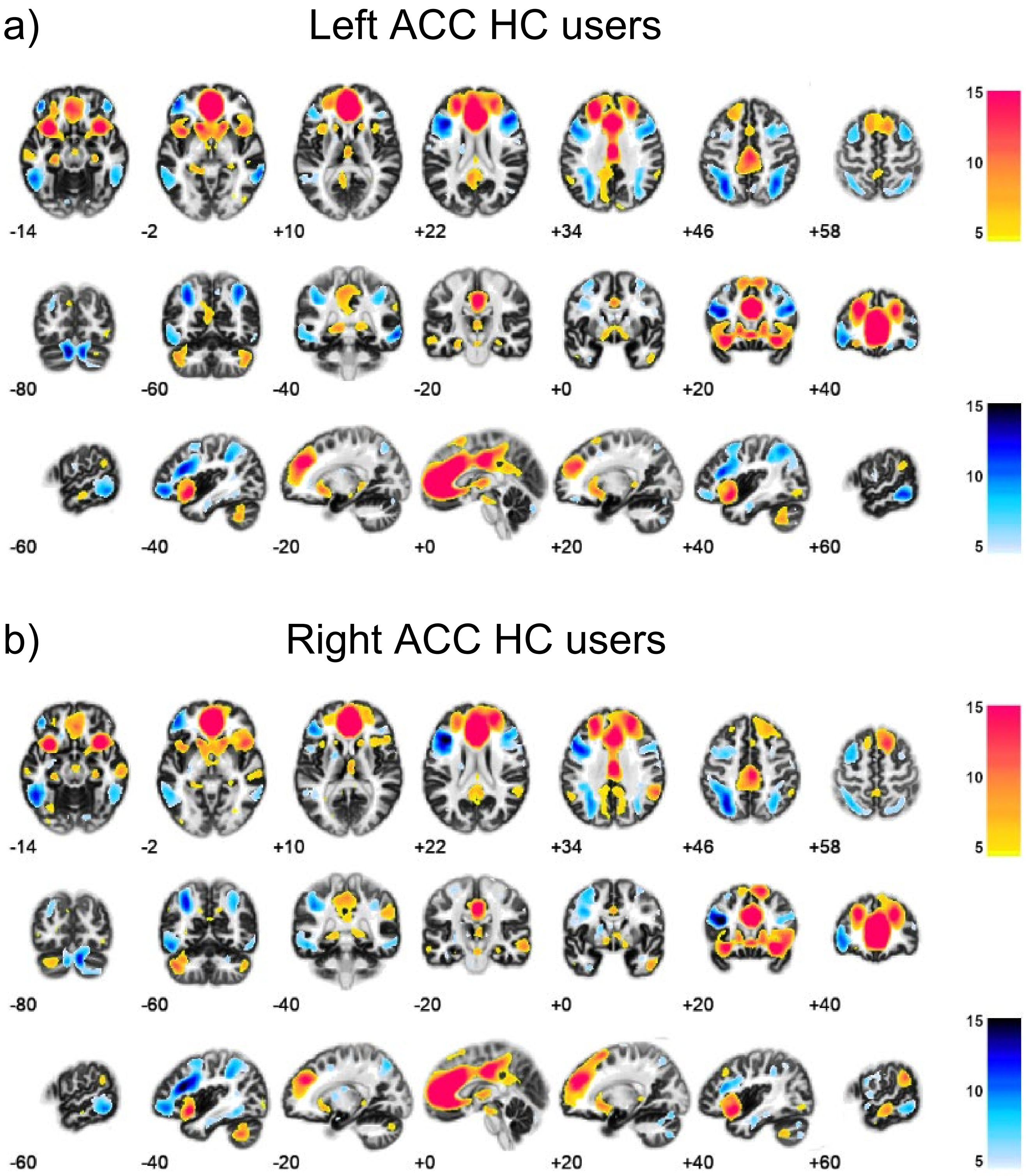

Figure S2. Seed to whole-brain connectivity for bilateral ACC in current HC users. Positive connectivity (in warm colours) and negative connectivity (in cold colours) from left and right ACC were in line with previous meta-analysis regarding these regions of interest. HC: hormonal contraceptive.

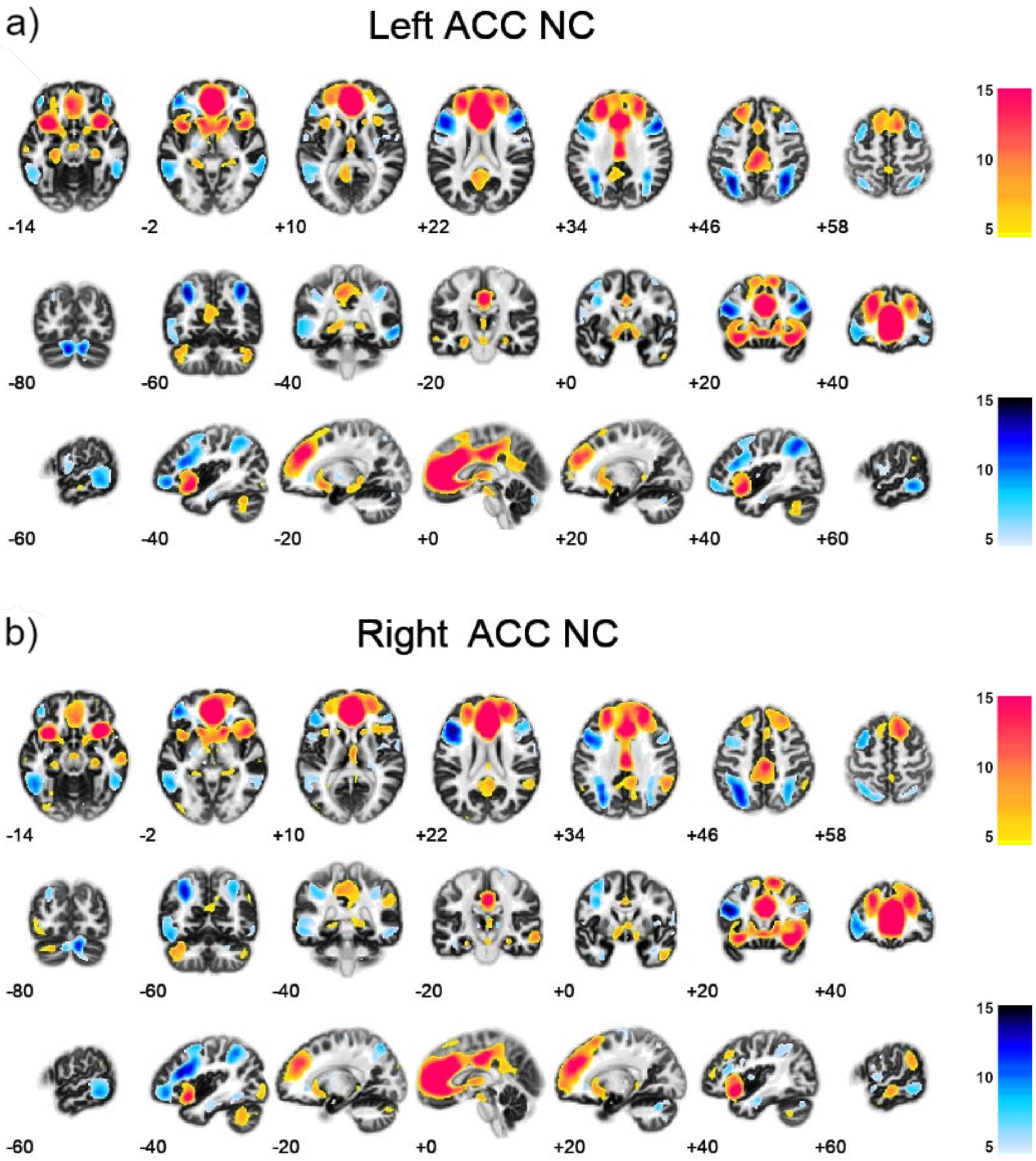

Figure S3. Seed to whole-brain connectivity for bilateral ACC in naturally cycling (NC) previous HC users. Positive connectivity (in warm colours) and negative connectivity (in cold colours) from left and right ACC were similar to current HC users, and in line with previous meta-analysis regarding these regions of interest.

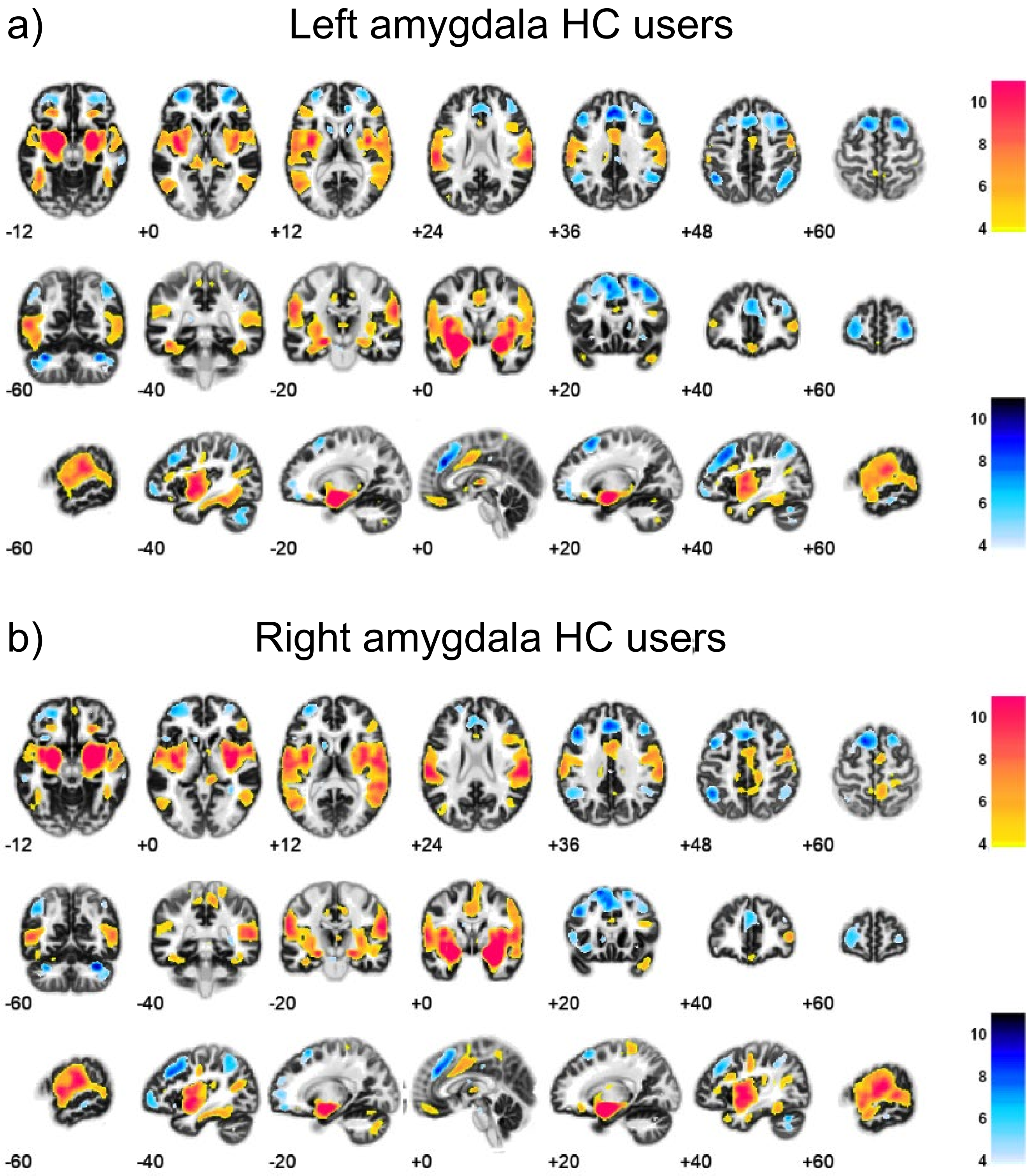

Figure S4. Seed to whole-brain connectivity for bilateral amygdalae in current HC users. Positive connectivity (in warm colours) and negative connectivity (in cold colours) from left and right amygdalae were in line with previous meta-analysis regarding these regions of interest. HC: hormonal contraceptive.

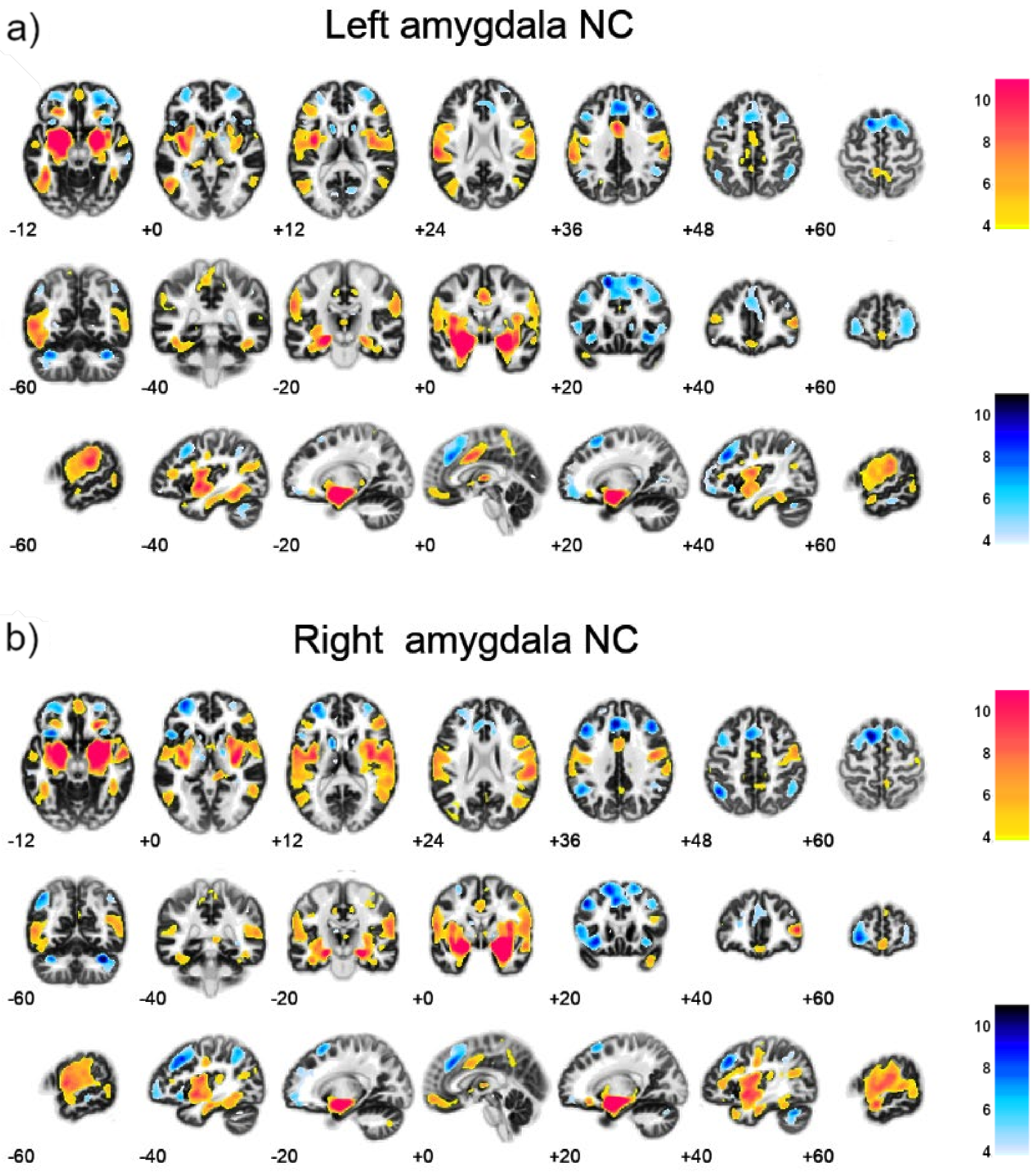

Figure S5. Seed to whole-brain connectivity for bilateral amygdalae in current naturally cycling (NC) previous HC users. Positive connectivity (in warm colours) and negative connectivity (in cold colours) from left and right amygdalae were similar to current HC users, and in line with previous meta-analysis regarding these regions of interest.

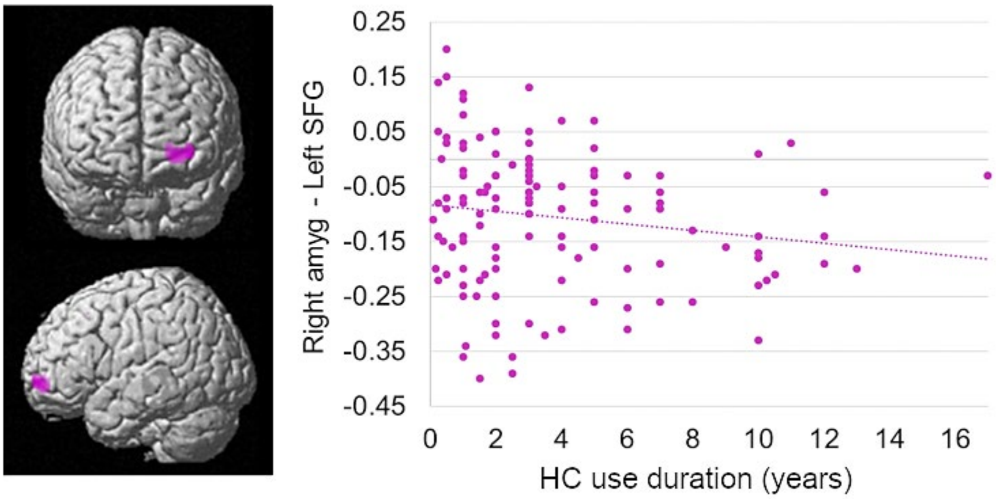

Fig S6. Main effect of previous HC use duration on right amygdala – left SFG connectivity. Independently of the androgenicity of the HC, previous users showed lower connectivity between right amygdala with left SFG the longer the use of the HC. HC: hormonal contraceptive.

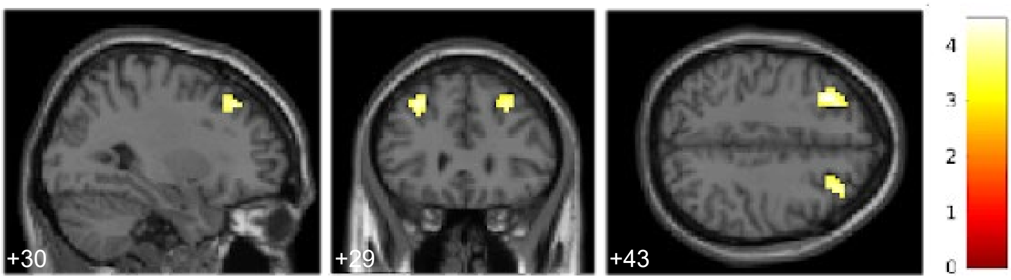

$p_{uncorrected}$	p_{FWE}	voxels	T	x,y,z	Area
<0.001	0.042	37	4.47	-21 56 -5	L SFG
<0.001	0.004	62	4.42	-30 26 46	L MFG
<0.001	0.081	31	3.99	30 32 43	R MFG

Fig S7. Main effect of current HC use duration on right amygdala – prefrontal connectivity. Independently of the androgenicity of the HC, previous users showed lower connectivity between right amygdala with left SFG and bilateral MFG the longer the use of the HC. HC: hormonal contraceptive.
